# Room temperature Co-doped manganite/graphene sensor operating at high pulsed magnetic fields

**DOI:** 10.1038/s41598-019-46012-2

**Published:** 2019-07-01

**Authors:** Rasuole Lukose, Nerija Zurauskiene, Voitech Stankevic, Milita Vagner, Valentina Plausinaitiene, Gediminas Niaura, Skirmantas Kersulis, Saulius Balevicius, Eleonora Bolli, Alessio Mezzi, Saulius Kaciulis

**Affiliations:** 1 Department of Functional Materials and Electronics, Center for Physical Sciences and Technology, LT-10257 Vilnius, Lithuania; 2 Department of Electrical Engineering, Faculty of Electronics, Vilnius Gediminas Technical University, LT- 10223 Vilnius, Lithuania; 3 Institute of Chemistry, Faculty of Chemistry and Geosciences, Vilnius University, LT- 03225 Vilnius, Lithuania; 4 Department of Organic Chemistry, Center for Physical Sciences and Technology, LT-10257 Vilnius, Lithuania; 5 Institute for the Study of Nanostructured Materials, ISMN - CNR, P.O. Box 10, Monterotondo, Rome Italy

**Keywords:** Materials for devices, Nanoscale materials

## Abstract

The demand to increase the sensitivity to magnetic field in a broad magnetic field ranges has led to the research of novel materials for sensor applications. Therefore, the hybrid system consisting of two different magnetoresistive materials – nanostructured Co-doped manganite La_1−x_Sr_x_(Mn_1−y_Co_y_)_z_O_3_ and single- and few-layer graphene – were combined and investigated as potential system for magnetic field sensing. The negative colossal magnetoresistance (*CMR*) of manganite-cobaltite and positive one of graphene gives the possibility to increase the sensitivity to magnetic field of the hybrid sensor. The performed magnetoresistance (*MR*) measurements of individual few layer (n = 1–5) graphene structures revealed the highest *MR* values for three-layer graphene (3LG), whereas additional Co-doping increased the *MR* values of nanostructured manganite films. The connection of 3LG graphene and Co-doped magnanite film in a voltage divider configuration significantly increased the sensitivity of the hybrid sensor at low and intermediate magnetic fields (1–2 T): 70 mV/VT of hybrid sensor in comparison with 56 mV/VT for 3LG and 12 mV/VT for Co-doped magnanite film, respectively, and broadened the magnetic field operation range (0.1–20) T of the produced sensor prototype.

## Introduction

Magnetoresistive (*MR*) sensors are widely used in daily applications^1^, for example, in information storage, where the data are received from a magnetic hard disk with *MR* read sensor that is very sensitive to low magnetic fields^2,3^. A group of materials, so called mixed-valence manganites, Ln_1−x_A_x_MnO_3_ (Ln = La, Nd, Pr, A = Ca, Sr, Ba, Pb)^4–7^ are the materials that have been intensively investigated for magnetic sensor applications due to the Colossal Magnetoresistance (*CMR*) phenomenon^8–10^. Indeed, manganite-based *MR* sensors, consisting of nanostructured thin film deposited on a polycrystalline substrate were already successfully developed^11^ measuring the magnitude of magnetic field independently of its direction (so-called *CMR*-B-scalar sensors)^12^. The demand for such *MR* sensors is increasing rapidly due to the development of advanced scientific and industrial devices and techniques such as non-destructive pulsed-field magnets, electromagnetic launchers, forming and welding, contactless high current measurement technique, etc. However, several existing problems concerning *MR* sensors have to be solved, like: (i) *MR* anisotropy (*MRA*) of nanostructured manganite films at fields <0.3 T (limited by demagnetization or ‘shape’ effect)^13^, (ii) sensitivity to magnetic field at temperatures higher than room temperature (limited by *Curie* temperature)^14^, and (iii) sensitivity at fields close to megagauss which is limited by *MR* saturation of manganites^11^. These problems are related to a very rich and complex physics of manganites (due to the electron-lattice and electron-electron interactions in these films) which then leads to the close relation of their structural, electronic and magnetotransport properties, therefore, the investigation of new manganite materials and innovative concepts for such sensors is of highest importance.

At this point, two-dimensional graphene has become a material of scientific and technological interest, due to remarkable electronic, mechanical, and thermal properties making graphene a highly attractive material for a large number of different applications^15,16^. Graphene’s properties are sensitive to the number of layers, their stacking configuration and misorientation to each other. The supporting substrate and number of defects or impurities influences the transport properties of graphene as well. Among various wonderful properties, graphene was reported to exhibit magnetoresistance phenomena^17,18^, implying the potential applications of this material in magnetic field sensing. However, the observed positive magnetoresistance of graphene, which is caused by Lorentz force induced Gauss effect, is small at low-field due to classical *MR*~*B*^2^ dependence^19^. In order to overcome the mentioned problems of manganite-based *MR* sensors and to increase the sensitivity of graphene in low magnetic fields, a novel hybrid graphene/manganite (GM) sensor based on combination of a single layer graphene (SLG) and La_0.82_Sr_0.18_MnO_3_ manganite film was recently proposed and investigated up to 2.3 T^20^. In the mentioned paper, the hybrid single layer graphene/manganite sensor showed the increased sensitivity in comparison to the individual graphene and manganite sensors. Therefore, further investigations concerning optimization of the magnetoresistive properties and the efforts to increase the sensitivity of these hybrid GM devices are of great interest.

In this study, the comprehensive investigations in magnetic fields up to 21 T of individual graphene structures consisting of few graphene layers and nanostructured manganite La_1−x_Sr_x_(Mn_1−y_Co_y_)_z_O_3_ films with partial Co substitution for Mn, as well as the results of a novel hybrid manganite-cobaltite/three-layer graphene sensor prototype are reported.

## Results and Discussion

### Single- and few-layer graphene: structural quality and magnetoresistance behavior

**Figure 1:**
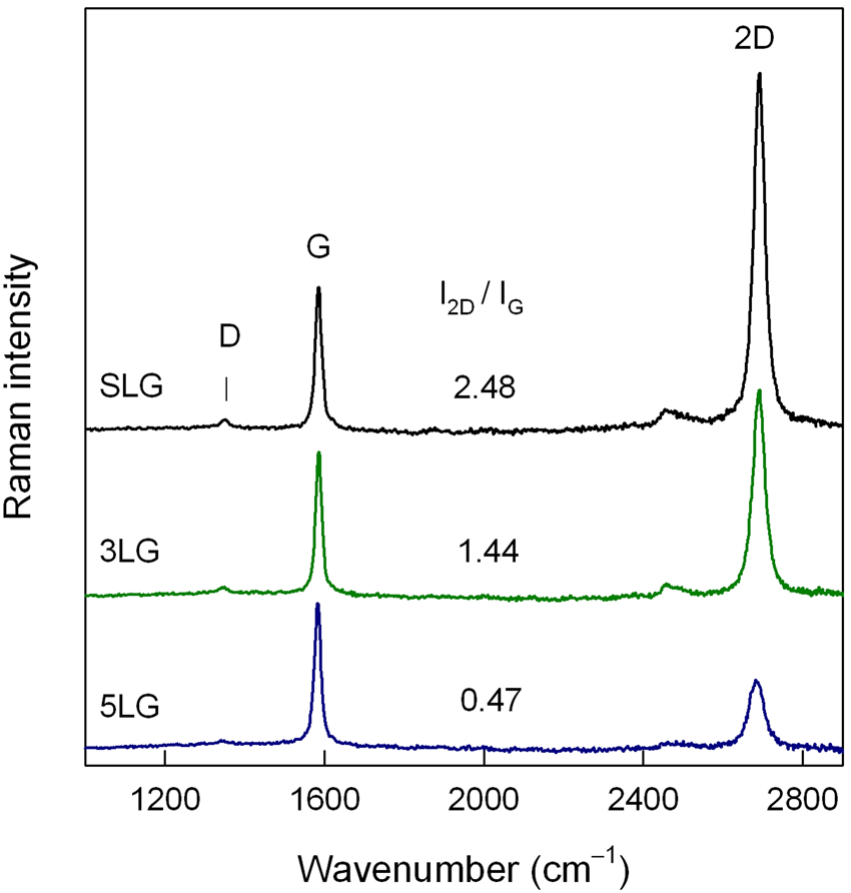
Resonance Raman spectra of exfoliated SLG, 3LG and 5LG graphene on Al_2_O_3_ substrate. Spectra are normalized to the intensity of G peak near 1585 cm^−1^. Excitation wavelength is 532 nm (0.6 mW).

Firstly, the structural quality of the prepared single and few-layers graphene was examined. The commercially available CVD grown single layer graphene (SLG) was transferred from Cu foil on Al_2_O_3_ substrate by wet chemical etching procedure (see Methods). A subsequent transfer of single layers of graphene and placement on top of another graphene layer resulted in few-layer graphene samples. The resonance Raman spectroscopy provides rich structural information on graphene-based materials^21–24^. It was demonstrated that the intensity ratio of the 2D band to G (*I*_2D_/*I*_G_) can be used to probe the number of graphene layers^25–27^. Figure 1 shows the resonance Raman spectra of graphene structures on Al_2_O_3_ substrate. The characteristic Raman feature for a single layer graphene is enhancement of *I*_2D_/*I*_G_ ratio (usually *I*_2D_/*I*_G_ > 2). For our studied samples the *I*_2D_/*I*_G_ ratios were found to be 2.48, 1.44, and 0.47 for SLG, 3LG, 5LG, respectively. This is consistent with previously reported data^25,28^. In addition, slight G peak frequency downshift was observed with increasing number of graphene layers indicating that *I*_2D_/*I*_G_ ratio trend is associated with changes in number of graphene layers^26^. The ratio *I*_D_/*I*_G_ sensitively probes the defects in graphene^21–23^. For our studied samples the intensity of D band was found to be very low (*I*_D_/*I*_G_ = 0.062 for single layer) indicating high structural quality of the graphene films.

**Figure 2:**
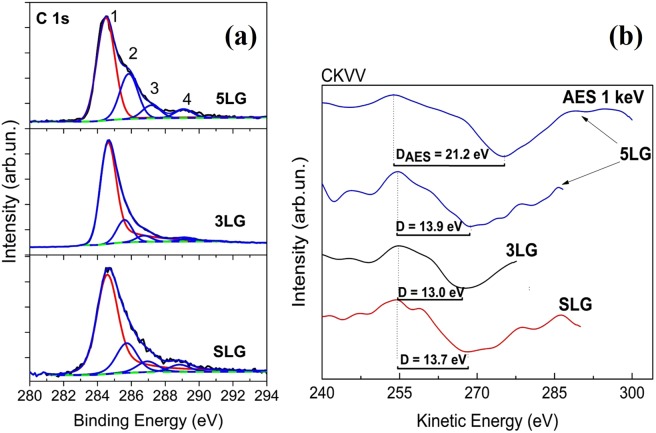
(**a**) Photoemission spectra of C 1 s region for the detection of PMMA polymer residuals in single- (SLG) few- (3LG, 5LG) layer graphene, after transfer process. The main synthetic component 1 is attributed to C−C bond in graphene; 2,3,4 components – to carbon bonds (−C−O, −C=O and −COOH) on the surface contamination including the residuals of PMMA polymer. (**b**) First derivative of XAES spectra of C KVV region for the samples SLG, 3LG, 5LG in order to prove existence of graphene in few-layer graphene samples. The AES spectrum of C KVV for the sample 5LG is also included.

However, the XPS analysis revealed the possible organic contamination with the increase of graphene layers. The residuals of PMMA polymer, used as support layer for the transfer of graphene, could explain the organic contamination which was confirmed by surface sensitive XPS measurements and Auger C KVV spectra. The main component 1 in the C 1 s photoemission spectra (see Fig. 2(a)) at binding energy BE = 284.6 eV corresponds to C−C bond in graphene, whereas the lower components 2, 3 and 4 at higher BEs are assigned to C−O, C=O and carboxylic bonds, respectively. The intensity of these components 2, 3 and 4 was strongly dependent on the surface cleaning procedure, indicating the possible presence of polymer residuals. The noticeably higher content of polymer residuals was detected in 5LG sample.

The characteristic D parameter determined from XAES measurements was in the range of 13.0−13.9 eV (see Fig. 2(b)) for almost all graphene samples and is in very good agreement with the literature data^29^. Despite the D parameter change to graphitic value of 21.2 eV for 5LG sample, the dominant presence of graphene is determined. XPS and XAES results revealed the existence of polymer traces with the increase of graphene layers, probably due to incompletely removed PMMA, captured at the interface between graphene layers.

**Figure 3:**
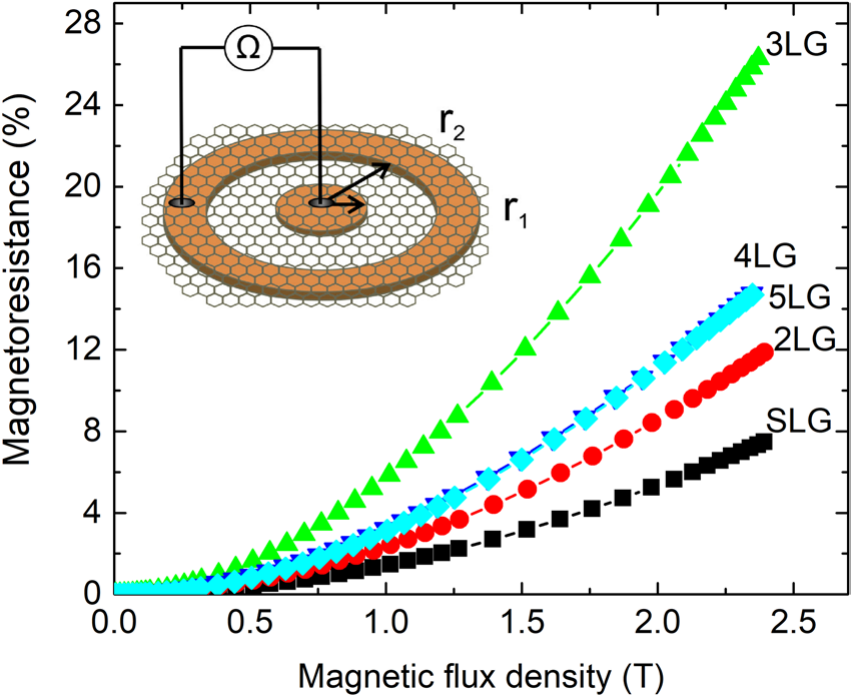
The magnetoresistance dependence on magnetic flux density at permanent magnetic field up to 2.35 T of single- and few-layer graphene at 300 K. The inset**:** Corbino disk configuration with graphene layer on top of Ag electrodes (orange) with the inner radius r_1_ = 0.7 mm, and the outer radius r_2_ = 2.3 mm. The current is passed through the disk-shaped area (white area) and the voltage difference is measured by application the magnetic field perpendicular to the surface.

The dependence of magnetoresistance *vs*. magnetic flux density for different number of graphene layers (1–5) is presented in Fig. 3. The *MR* behavior of graphene samples was investigated using Corbino disk configuration (see inset in Fig. 3) with radiuses of *r*_1_ = 0.7 mm, *r*_2_ = 2.3 mm). This geometry eliminates any Hall voltage contribution and allows achieving the maximal values of longitudinal magnetoresistance (caused by Gauss effect) in graphene. The manetoresistance was determined from the following equation at a certain magnetic field (magnetic flux density *B*): *MR*(%) = [*(R(B)*-*R(0))*/*R(0)*] × 100%, where *R(0)* – resistance in zero magnetic field and *R(B)* – resistance in magnetic field. The observed *MR* values subsequently increased for the SLG, 2LG and 3LG and started to decrease for 4LG, 5LG samples, in the investigated magnetic field range (Fig. 3). The influence of the number of graphene layers on magnitude of *MR* could be explained by the coupling between the layers leading to the changes of physical properties. Compared to the strong sp^2^ bonding between the carbon atoms in the SLG sheet the weak van der Waals forces between the graphene layers allows the formation of different hybridized n-layered configurations. The resulting system is then different from both 2D and 3D systems (graphene and graphite, respectively) and depends strongly on the number of layers, on the stacking sequence (ABA, ABC for few graphene layers)^30,31^ or stacking in rotational disorder between the placed graphene sheets, resulting in turbostratic graphite. The significant influence of graphene stacking sequence on the properties of the system was noticed in the field-effect transistor configuration which was used with changeable gate voltage^30,31^. Liao *et al*.^32^ observed the reduction of *MR* of 3LG in comparison with 2LG, with the current applied perpendicularly to graphene. The authors obtained inconsistent decrease of the *MR* with the increase of number of graphene layers (up to 10) and explained it by decrease of charge carrier mobility evaluated from quadratic magnetoresistance dependence on magnetic flux density *MR*~*B*^2^. In our study, in comparison to ref.^32^ the current flows in-plane. However, the change of carrier mobility could be also the most probable reason for the *MR* decrease in multi-layer graphene. We propose, that the grain boundaries/impurities (due to graphene growth and transfer processes) in graphene are acting like scattering centers for charge carriers and reduce their mobility. The vertical transport of charge carriers at graphene/graphene interface allows to “overcome” different inhomogeneities and leads to the reduced electrical resistance and increased magnetoresistance in the few-layer graphene structure. The carrier mobility (*µ*) values in graphene layers were extracted from the fitting the quadratic part of *MR* dependences of the experimental curves (*MR*~(*μB*)^2^) presented in Fig. 3 . The measured carrier mobilities for graphene samples (n = 1–5) differ in the following way: μ = 1180 cm^2^/Vs for SLG; 1530 cm^2^/Vs for 2LG; 2420 cm^2^/Vs for 3LG; 1790 cm^2^/Vs for 4LG and 1780 cm^2^/Vs for 5LG. It is obvious, that three-layer graphene exhibits highest mobility values. High *μ* values could be explained by small number of defects which is confirmed by intensity of the D-band in Raman spectra (see Fig. 1). In comparison with the results of Liao *et al*.^32^, where the highest μ ≈ 540 cm^2^/Vs at 2 K and ≈310 cm^2^/Vs at 300 K were observed, the mobilities of our samples are much higher. The reduction of *MR* in 4LG and 5LG samples could be probably related to the limited mobility of the charge carriers due to the higher content of PMMA residuals as observed from XPS study (see Fig. 2(a)), as well as to the formation of inhomogeneities (wrinkles, ripples) during the repetitive graphene transfer procedure.

**Figure 4:**
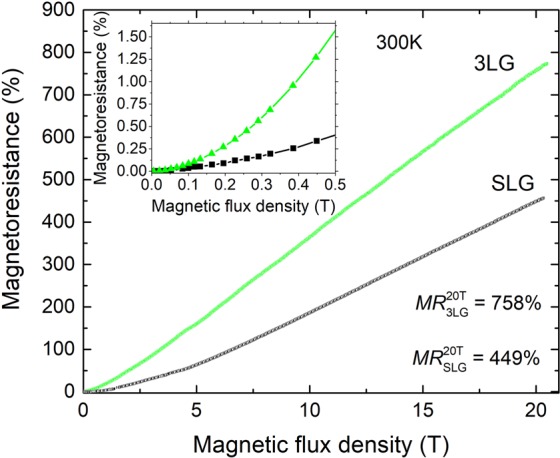
The magnetoresistance dependence on magnetic flux density at pulsed magnetic field up to 21 T of single- and three-layer graphene at 300 K. The inset: The magnetoresistance dependence on magnetic flux density up to 0.5 T for single- and three-layer graphene.

A quadratic *MR* dependence on magnetic flux density *B* was observed by several authors as usual behavior for semiconductor materials at low magnetic fields^32–34^. However, creating inhomogeneities or with applied nanoscale manipulation (functionalization of graphene, combination of graphene with different type of substrate materials like boron nitride, black phosphorus, and etc.) it is possible to induce a large non-saturating linear magnetoresistance, desirable property for practical applications^32^. Linear *MR* has been observed in multilayer graphene^35^, in exfoliated^36^ and in epitaxial bilayer graphene^19^ up to 62 T. Our study agrees well with the literature data since we also observe linear magnetoresistance dependence on magnetic flux density. The interesting point is that the region of quadratic (*B*^2^) dependence of magnetoresistance differs depending on the number of the stacked graphene layers. In our experiments, in the sequence of SLG, 2LG and 3LG, the quadratic part of *MR* decreases (*MR*~*B*^2^ is fair up to 1.7 T for SLG, up to 1 T for 2LG and up to 0.7 T for 3LG) and the linear part starts at lower and lower magnetic field. The reduction of quadratic *MR* could be explained by the interlayer tunneling of charge carriers in few layer graphene in comparison with SLG^37^. For the multi-layer graphene (4LG and 5LG) the quadratic parts increase again in comparison with 3LG up to 1 T, but still has linear dependence of the *MR* with higher values in comparison with SLG (Figs 3 and 4). As the number of the layers increases, the vertical transport of charge carriers also becomes possible, and the scattering from the interfaces could play a significant role. Therefore, the change of the *MR* behavior from quadratic to linear dependence, with a number of graphene layers, is related to several competing processes mentioned above. The main observation of our study is that 3LG has the narrowest *B*^2^ part and the highest linear *MR* in all investigated magnetic field range. This property broadens the application range of the possible magnetic field sensor based on 3LG structure, making it quite unique because of the high sensitivity at low and high magnetic fields.

According to the observed *MR* results at intermediate (up to 2.3 T) magnetic field, the single- and three-layer graphene were further investigated in pulsed magnetic fields up to 21 T. The experiments revealed that room temperature *MR* of three-layer graphene increased by 2 times (up to 758%) at 21 T (Fig. 4) in comparison with SLG (*MR* = 450%). Moreover, the 3LG showed the *MR* increase in all investigated range starting already from very low magnetic field of 0.1 T.

In comparison to the CVD grown few-layer graphene, the *MR* of 60% at 14 T magnetic field and at room temperature was reported^32^. In our study, the *MR* of 300%, at the same 14 T magnetic field, was achieved for transferred SLG. Nevertheless, one has to be aware by comparing the results of different groups since the *MR* values depend on the growth/production method of graphene, number of the graphene layers, supporting substrate and configuration of the measurement system^15^. In the case of 2LG graphene Kisslinger and others^19^ observed *MR* of about 800% at 21 T and 300 K, which is in a good agreement with our results. Additionally, our results demonstrate that magnetic field sensor consisting of 3LG (instead of SLG) could lead to higher sensitivity to the magnetic field in the range of [0.1−20 T].

### Magnetoresistance of nanostructured La_1−x_Sr_x_(Mn_1−y_Co_y_)_z_O_3_

**Figure 5:**
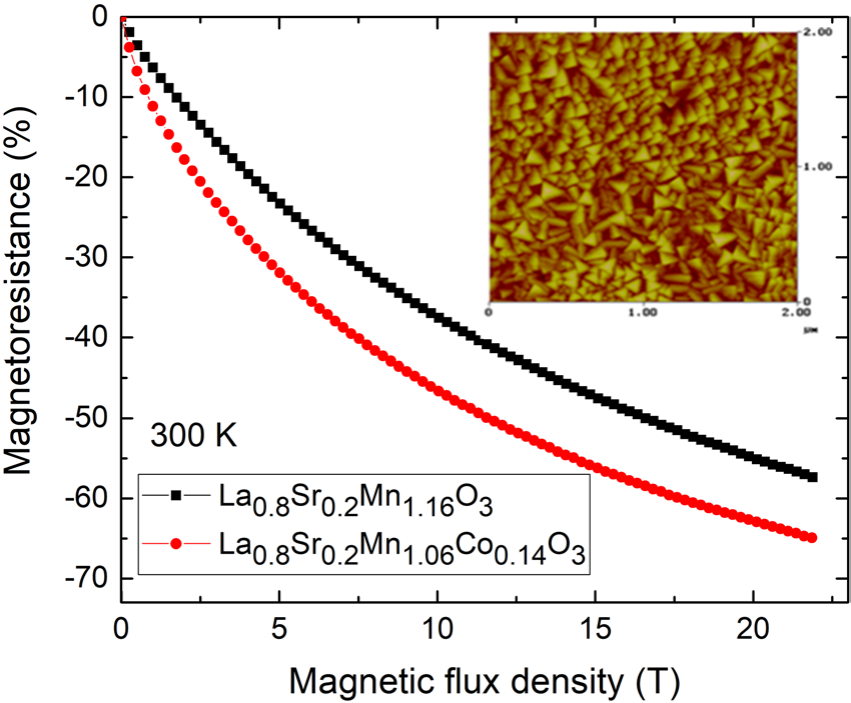
The magnetoresistance magnitude dependence on magnetic flux density of nanostructured Co-doped manganite films at 300 K. The inset: The typical surface morphology of 360 nm thick LSMCO films taken by Atomic Force Microscopy in tapping mode.

Previously, it was demonstrated, that the lanthanum manganites exhibit high magnetoresistance values at relatively low magnetic fields^38,39^ and have tendency of saturation at high magnetic fields^11^. Therefore, the idea to combine two materials (graphene and manganite) having different magnetoresistance behavior was realized. In order to increase the *MR* of lanthanum manganite (La_1−x_Sr_x_MnO_3_), the La_1−x_Sr_x_(Mn_1−y_Co_y_)_z_O_3_ (LSMCO) films were grown by Pulsed-Injection Metal-Organic Chemical Vapor Deposition (PI MOCVD) technique on polycrystalline Al_2_O_3_ substrate. The films grew either by layer-then-island or by island growth mechanisms resulting in the polycrystalline structure with the crystallite size varying from 70 up to 120 nm (nanostructured film) and surface roughness of 18−19 nm (Fig. 5-inset). The TEM images (not shown) demonstrated nanostructured films with column-like crystallites spread through the entire thickness of the films perpendicular to the substrate plane^40^. Such nanostructured films have an advantage in comparison with the epitaxial ones, since the magnetic sensors produced using these films operate in a broader temperature and magnetic field range^41^. It was demonstrated that La_1−x_Sr_x_MnO_3_ films doped with a certain amount of Co (y = 0.06–0.08) exhibit higher resistivity and magnetoresistance values at room temperature^42,43^ thus, the substitution of Co for Mn in manganite films could increase the sensitivity of the sensors to magnetic field. Figure 5 presents the magnetoresistance dependences on magnetic flux density for two films: LSMO and LSMCO doped with Co (y = 0.14), exhibiting negative colossal magnetoresistance phenomenon. The magnetoresistance of manganite films was determined by the same formula as for graphene layers: *MR*(%) = [*(R(B)* − *R(0))/R(0)*] × 100%. Acording to our results, the certain content of Co (y = 0.14) increased the *MR* from 55% to 63% at 21 T magnetic field in comparison with undoped manganite film.

The increase of *MR* was observed in the whole range of investigated magnetic field, showing the increase of sensitivity to magnetic field for Co-doped manganites not only at high (up to 21), but also at low magnetic fields (lower thant 2 T). The dopping at so-called B-site (Co substitution for Mn), destroys the long range ferromagnetic ordering and Mn^3+^-O-Mn^4+^ network, resulting in the change of electric and magnetic properties. Thus, the Co substitution for Mn results in the decrease of the transition temperature from the paramagnetic to ferromagnetic phase and increase of the resistivity, resulting in the increase of room temperature magnetoresistance^42,43^. However, to compare *MR* values of manganite-cobaltite films and *MR* of graphene layers is complicated and not straightforward. As follows, in manganite-cobaltite case, the zero field resistance (*R(0)*) was the largest at zero magnetic field due to the negative *MR*, while it was smallest one for graphene, due to positive *MR* effect. Therefore, the *MR* values of manganite-cobaltite films were always less than 100% (Fig. 5), while for graphene the *MR* can reach hundreds of % (Fig. 4).

### The LSMCO/3LG sensor prototype for operation in high pulsed magnetic fields

**Figure 6:**
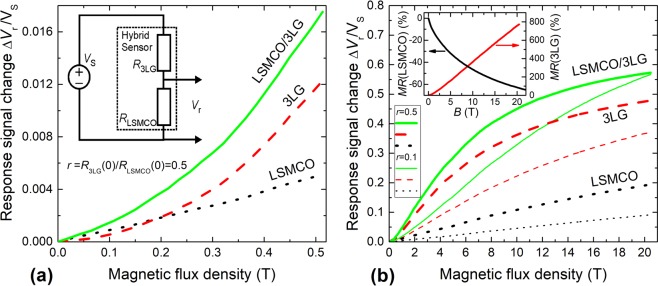
The response signal change (*ΔV*_*r*_/*V*_S_) dependence on magnetic flux density in pulsed magnetic field up to 0.5 T **(a)** and 20 T **(b)** of LSMCO, 3LG and hybrid LSMCO/3LG sensor at 300 K, when the ratio of zero field resistances of individual elements *r* = *R*_3LG_(0)/*R*_LSMCO_(0) were equal to 0.5 and 0.1. The (a-inset): A voltage divider configuration used for the measurements in pulsed magnetic field. The (b-inset): the *MR* dependence on magnetic flux density of individual LSMCO and 3LG elements.

In order to increase the sensitivity of graphene at low magnetic fields, the hybrid sensor was designed (see inset in Fig. 6(a)). It combined the 3LG graphene and manganite-cobaltite film connected in voltage divider configuration. In this case, the graphene layers were transferred on Al_2_O_3_ substrates with Ag contacts in Corbino disk configuration and connected in series with manganite-cobaltite film. The sensor was supplied by constant voltage *V*_S_ = 1.25 V and the voltage drop across the LSMCO film was measured. In order to obtain the convenient voltage drop across the magnetic field sensor, the current flowing through the structure during measurements was in the range of 1–3 mA. It has to be noted, that the investigations of reduced graphene oxide films^44^ revealed the dependence of magnetoresistance on the supplied current. However, this dependence was observed only in the range of low currents (*I* < 10 µA). In our case, the current was much higher and therefore no dependence of the *MR* on the flowing current magnitude was observed for various graphene layers having different resistances.

The normalized response signal *ΔV*_*r*_/*V*_S_ of individual elements (LSMCO, 3LG) and hybrid LSMCO/3LG sensor at magnetic fields up to 0.5 T is presented in Fig. 6(a), where the voltage change *ΔV*_*r*_ is normalized to the source voltage *V*_S_: *ΔV*_*r*_/*V*_S_ = (*V*_*r,B=*0_ − *V*_*r,B*_)/*V*_S_, *where V*_*r,B=*0_ – response voltage without magnetic field and *V*_*r,B*_ – at magnetic field, respectively. The individual elements (LSMCO and 3LG) were connected in series with the ballast resistors in the same voltage divider configuration, by replacing other element (LSMCO or 3LG) with ballast resistor, respectively, depending on the investigated element (3LG or LSMCO). In order to compare the response of individual elements with the hybrid device, the ballast resistors with the same values as zero field resistance of the other element in the hybrid structure, were used for the measurements. The measurements revealed, that in magnetic fields up to 0.2 T the response signal of individual 3LG was lower than signal of individual LSMCO sample: e. g. at magnetic field of *B* = 0.07 T it differs more than two times. This could be attributed to different *MR* behavior of both materials at low magnetic fields: quadratic dependence of graphene and almost linear of LSMCO. Therefore, the connection of 3LG and LSMCO in voltage divider geometry lead to increased sensitivity of the device, in particular, at the low magnetic field range. This effect could be explained by different signs of magnetoresistance - positive *MR* of graphene and negative *MR* of nanostructured manganite-cobaltite film (see inset in Fig. 6(b)) – where the resistances of these components changes in opposite way with applied magnetic field. As a result, the fraction of voltage drop across manganite-cobaltite film increases due to resistance change of graphene.

Additionally, the response signals of hybrid sensor and individual samples in pulsed magnetic fields up to 20 T is presented in Fig. 6(b): two cases *r* = 0.5 and *r* = 0.1 are shown, where *r* is a ratio of zero field resistances of individual elements *r* = *R*_3LG_(0)/*R*_LSMCO_(0). It is obvious, that hybrid LSMCO/3LG sensor exhibits significantly higher response signal in all range of magnetic field in comparison with individual elements. However, depending on the ratio *r*, one can tune the highest response signal of the hybrid sensor and its sensitivity to the lower or higher magnetic field ranges. The normalized sensitivity of the hybrid sensor was evaluated as *S* = (δΔ*V*_*res*_/δ*B*)/*V*_*S*_. The sensitivity values of individual 3LG, LSMCO elements and the hybrid LSMCO/3LG sensor (determined from Fig. 6) at different magnetic field ranges are listed in Table 1. The highest sensitivity 70 mV/VT of the hybrid sensor can be achieved for the case *r* = 0.5 in the range of 1–2 T and it is high enough at lower fields: 57 mV/VT at 0.5 T (in comparison, 12 mV/T and 40 mV/T for individual 3LG, LSMCO, respectively). These results demonstrate improvement of sensitivity of Co-doped manganite/3-layer graphene sensor in comparison with previously published results on manganite/single layer graphene sensor^20^ exhibiting normalized sensitivities of 16 mV/VT at 0.5 T and 29 mV/VT at 2 T. However, at 20 T the sensitivity of the LSMCO/3LG sensor drops to 7 mV/VT for *r* = 0.5, respectively. It has to be noted that in the case of *r* = 0.1 the sensitivity of hybrid sensor is lower at low fields (20 mV/VT at 0.5 T), however, it is high enough in a wide range of magnetic fields (40 mV/VT at 2.5–7 T and 17 mV/VT at 20 T) and no saturation of the response signal was observed up to 20 T (see Fig. 6(b)). It is difficult and not completely correct to compare the sensitivities of the developed hybrid magnetic field sensor and other type sensors described in the literature, due to the different magnetic field operation ranges and sensor configuration geometries. For instance, it was obtained for the silicon - based Hall sensor *S* = 96 mV/VT and graphene-based Hall sensor (35–3000) mV/VT^45,46^, however mentioned sensors were operating only in mT range of permanent magnetic field.

**Table 1:** Sensitivity of individual elements LSMCO, 3LG and hybrid sensor LSMCO/3LG at different magnetic field ranges for *r* = 0.5 and *r* = 0.1, where *r* is a ratio of zero field resistances of individual elements (*r* = *R*_3LG_(0)/*R*_LSMCO_(0)).

Sensor	*S*, mV/(V*T) (at *B* = 0.5 T)	*S* maximal, mV/(V*T) (field range *B*)	*S*, mV/(V*T) (at *B* = 20 T)
**LSMCO** R_bal_ = R_3LG_(0) R_bal_/R_LSCMO_(0) = 0.5	12	12 (*B* = 0.3 ÷ 10 T)	7
**3LG** R_bal_ = R_LSCMO_(0) R_3LG_(0)/R_bal_ = 0.5	40	56 (*B* = 1 ÷ 2 T)	7
**Hybrid** **R**_**3LG**_ **(0)/R**_**LSMCO**_ **(0) = 0.5**	**57**	**70** **(** ***B*** **= 1 ÷ 2 T)**	**7**
**LSMCO** R_bal_ = R_3LG_(0) R_bal_/R_LSCMO_(0) = 0.1	5	5 (*B* = 0.3 ÷ 20 T)	5
**3LG** R_bal_ = R_LSCMO_(0) R_3LG_(0)/R_bal_ = 0.1	17	27 (*B* = 2.5 ÷ 7 T)	10
**Hybrid** **R**_**3LG**_ **(0)/R**_**LSMCO**_ **(0) = 0.1**	**21**	**40** **(** ***B*** **= 2.5 ÷ 7 T)**	**7**

In conclusion, the obtained results show the potential application of Co-doped manganite/3LG sensor for magnetic field measurement application at room temperature in very broad magnetic field range [0.1−20 T] with a certain sensitivity values depending on the magnitude of magnetic field. It has to be noted, that for the device fabrication the sensitivity dependence on the magnetic field can be solved by modern electronics with in advance stored calibration data, converting the measured signals into magnetic field values^12^. Additionally, LSMCO/3LG sensor could be also used at lower operation temperatures since three-layer graphene exhibits even higher *MR* at cryogenic temperatures (914% at 100 K, 21 T).

## Methods

The commercially available, high quality monolayer graphene grown by CVD method on Cu foil was used for the experiments. The single graphene layer grown on Cu foil was transferred to target substrate by applying wet chemical etching procedure. During this procedure Cu substrate was etched by ammonium persulfate solution from the bottom, leaving a floating graphene/PMMA flake on the surface of etching solution. Afterword, the SLG + PMMA flake was rinsed several times in deionized water and transferred to the target substrate (Al_2_O_3_) with formed Ag contacts in Corbino disk configuration for electric and magnetotransport measurements. PMMA polymer is used as supporting and protecting layer from folding and physical damages of graphene layer. The same transfer procedure was applied in order to get few-layer graphene samples, only the PMMA layer was removed each time with acetone and iso-propanol before placing the next graphene layer on the top of the previous one.

The structural quality and number of graphene layers were confirmed by Raman spectroscopy. Raman spectra were recorded using a confocal spectrometer/microscope inVia (Renishaw, UK) equipped with thermoelectrically cooled (−70 °C) CCD camera and microscope. Spectra were excited with continuous-wave diode-pumped solid state laser providing 532 nm radiation. The 50x/0.75 NA objective lens and 1800 lines/mm grating were used to record the spectra. The accumulation time was 50 s. To avoid damage of the sample, the laser power at the sample was restricted to 0.6 mW. The laser beam was focused to ~2 μm diameter spot on the sample. Raman scattering wavenumber axis was calibrated by the polystyrene Standard Raman spectrum. Parameters of the bands were determined by fitting the experimental spectra with Gaussian-Lorentzian shape components using GRAMS/A1 8.0 (Thermo Scientific) software.

X-ray photoemission (XPS) and X-ray induced Auger (XAES) spectra were collected by using an Escalab 250Xi (Thermo Fisher Scientific Ltd., East Grinstead, UK) spectrometer with a monochromatic Al Kα excitation source and six-channeltron detection system. The photoemission spectra were collected in standard mode of electromagnetic lenses from the area of about 1 mm in diameter at 40 eV pass energy of the analyzer. For Auger electron spectroscopy (AES) was employed an Escalab MkII spectrometer (VG Scientific Ltd., East Grinstead, UK) with electron gun LEG200 operated at low energy (1–2 keV) and low beam current (few nA). All C KVV spectra (XAES and AES) were acquired at 100 eV pass energy and later smoothed by moving average algorithm for 11 times with a width of 1.8 eV. Afterwards, these spectra were differentiated by using a width of 7 data points for the determination of D parameter^47^ of carbon. Spectroscopic data were processed by the Avantage v.5 software (Thermo Fisher Scientific Ltd.).

The nanostructured La_1−x_Sr_x_(Mn_1−y_Co_y_)_z_O_3_ (LSMCO) films were grown on polycrystalline Al_2_O_3_ substrates by pulsed-injection MOCVD technique with supplying the mixture of precursor solution in certain micro-doses^48^. The 360 nm thick LSMCO films were deposited at 750 °C temperature and 10 Torr pressure with a partial 3.5 Torr oxygen pressure. The Co doping level in the films (y = 0.14) was chosen in order to achieve higher magnetoresistance values of the films at room temperatures in comparison with manganite films without Co doping. Other deposition parameters (temperature, pressure, growth rate, and etc.) were controlled and optimized in order to get LSMCO films with a certain column-like nanostructure responsible for high magnetoresistance values in a wide temperature and magnetic field range.

The morphology of the films was investigated using Atomic Force Microscopy (Veeco Multi-Mode Nanoscope II) (AFM) and Scanning Electron Microscopy (Hitachi SU70) (SEM). The nanostructure of the films was studied using Transmission Electron Microscopy (Tecnai G2 F20 X-TWIN) (TEM).

For the magnetoresistance measurements of manganite-cobaltite films the traditional photolithography and mesa etching was applied to form rectangular shaped LSMCO films. The Ag electrodes with a Cr sublayer were thermally deposited over the LSMCO films and post annealed at 450 °C for 1 h in O_2_ atmosphere. The distance between electrodes was constant and equal to 50 μm, whereas the width of the sample was changed to match the zero field resistance ratio between graphene and LSMCO samples.

The resistivity and *MR* measurements of manganite-cobaltite and graphene samples were performed at 300 K in permanent magnetic field up to 2.35 T by using electromagnet. The mobile 43 kJ pulsed magnetic field generator was used for investigation of the samples in pulsed magnetic field up to 21 T. The generator was capable to generate pulsed magnetic fields of half-sine shape with amplitudes up to 40 T and pulse durations in the range of 0.3–2 ms depending on the chosen inductor (fabricated in the laboratory). The high pulsed magnetic field generator consists of high voltage power supply, capacitor bank, high power switch and a special multi-shot magnetic field coil (inductor). The high-field facilities were designed and are used for the investigations of properties of different materials in high magnetic fields at the Center for Physical Sciences and Technology in Vilnius, Lithuania^49^.

## Conclusions

In the present study, the quality and magnetoresistance properties of graphene layers (n = 1–5) were measured. The Raman measurements confirmed the good quality of graphene samples, whereas the *MR* measurements showed the linear dependence of magnetoresistance for all graphene structures reaching the highest *MR* values of 758% at 21 T for the three-layer graphene (two times higher as single one). The revealed highest response of 3LG to magnetic field lead to the development of hybrid magnetic sensor prototype consisting of Co-doped manganite/three-layer graphene system for the sensing applications at room temperature. The prototype sensor demonstrated that combination of materials with positive (graphene) and negative (Co-doped manganite) magnetoresistance effects leads to a significant increase of the sensitivity (2 times in comparison with 3LG at 0.1 T, exhibiting the largest sensitivity of 70 mV/VT at 1–2 T) and broadens the magnetic field operation range from 0.1 up to 20 T. Moreover, the possibility to tune the sensitivities depending on the magnetic field range during the fabrication stage, by changing the ratio of zero field resistances of 3LG and LSMCO, was demonstrated.

## Data Availability

There are no any restrictions related to the data availability.
